# *Polygonum cuspidatum* inhibits pancreatic lipase activity and adipogenesis *via* attenuation of lipid accumulation

**DOI:** 10.1186/1472-6882-13-282

**Published:** 2013-10-25

**Authors:** Young Sook Kim, Yun Mi Lee, Joo Hwan Kim, Jin Sook Kim

**Affiliations:** 1 Korean Medicine-Based Herbal Drug Development Group, Herbal Medicine Research Division, Korea Institute of Oriental Medicine (KIOM), Daejeon 305-811, Republic of Korea; 2 Department of Life Science, Gachon University, Seongnam, Kyonggi-do 461-701, Republic of Korea

**Keywords:** Adipocyte differentiation, Phosphorylated AMP-activated protein kinase (pAMPK), Adipocyte differentiation-related protein (ADRP), Perilipin, PPAR-gamma, C/EBP-alpha, *Polygonum cuspidatum*

## Abstract

**Background:**

Obesity causes metabolic disease and is a serious health problem around the world. *Polygonum cuspidatum* (POCU1b) has been used clinically for the treatment of constipation, gallstones, hepatitis, and inflammation in East Asian countries. The principal aim of this study was to investigate for the first time whether the extract of *Polygonum cuspidatum* (POCU) biologically affects adipogenesis in 3 T3-L1 preadipocytes.

**Methods:**

Fractions (*n*-hexan, ethyl acetate, *n*-butanol, and water) of POCU ethanol extract were evaluated *in vitro* for their inhibitory activities on pancreatic lipase. Of the fractions, the *n*-butanol of POCU ethanol extract (POCU1b) was examined anti-obesity activity in 3 T3-L1 preadipocytes. To examine the inhibitory effect of POCU1b on adipogenesis, 3 T3-L1 preadipocytes were treated every the other day with POCU1b at various concentrations (0 ~ 25 μg/mL) for twelve days. Oil-red O staining and triglyceride content assay were performed to determine the lipid accumulation. The expression of mRNA and proteins associated lipid accumulation was measured using RT-PCR and Western blotting analysis. We also examined the effect of POCU1b on level of phosphorylated AMP-activated protein kinase (pAMPK) in 3 T3-L1 preadipocytes with POCU1b at various concentrations during adipocyte differentiation.

**Results:**

POCU1b exhibited the most pronounced inhibitory effects on pancreatic lipase activity. We found that POCU1b inhibited adipocyte differentiation in 3 T3-L1 preadipocytes in a dose-dependent manner, as evidenced by the reduced formation of lipid droplets and decreased glycerol-3-phosphate dehydrogenase (GPDH) activity. We also showed that the expression levels of adipocyte differentiation-related protein (ADRP) and perilipin (a protein that coats lipid droplets in adipocytes) were both reduced after POCU1b treatment. Peroxisome proliferator-activated receptor-gamma (PPAR-γ) and CCAAT/enhancer-binding protein-alpha (C/EBP-α) proteins, both major adipogenic transcription factors, were markedly reduced by POCU1b. Moreover, ADRP, perilipin, C/EBP-α, and PPAR-γ mRNA levels were also reduced by POCU1b. Levels of phosphorylated AMP-activated protein kinase (pAMPK) were elevated after POCU1b treatment (5, 10, and 25) in a dose-dependent manner.

**Conclusions:**

Taken together, these results suggest that the anti-obesity effects of POCU1b involve the inhibition of pancreatic lipase activity and adipogenesis *via* the down-regulation of lipid accumulation.

## Background

Obesity, a common nutritional disorder, results from the disequilibrium between energy intake and expenditure. It is believed to be associated with numerous diseases, including hyperlipidemia, hypercholesterolemia, hypertension, and type 2 diabetes [1,2]. Adipocytes store excess energy in the form of triglycerides which are contained inside lipid droplets, organelles composed of a neutral lipid core surrounded by a protein-coated single phospholipid layer [3]. Both the number and size of mature adipocytes are increased compared with preadipocytes in adipose tissues, and the inhibition of adipogenesis during the maturation of preadipocytes to adipocytes regulates the amount of adipose tissue mass [4].

Natural products have the potential to inhibit adipogenesis and induce the apoptosis of mature adipocytes [5,6]. Herbal extracts and phytochemicals have shown anti-obesity effects, especially the down-regulation of the adipogenic transcriptional factors peroxisome proliferator-activated receptor-γ (PPAR-γ) and CCAAT/enhancer binding protein-α (C/EBP-α), the inhibition of fatty acid accumulation, and the stimulation of glycerol-3-phosphate dehydrogenase (GPDH) activity in 3 T3-L1 preadipocytes [7-10]. Berberine, a natural plant product, displays beneficial effects in the treatment of diabetes and obesity at least in part via the stimulation of AMP-activated protein kinase (AMPK) activity [11]. AMPK is an intracellular energy sensor that plays a central role in the regulation of lipid and glucose metabolism [12]. Hydroxycitric acid (HCA), the active ingredient in the herbal compound Garcinia cambogia, acts as an anti-obesity agent and has been used as a natural supplement for weight management [13]. Recently, newer approaches for the treatment of obesity have involved inhibition of dietary triglyceride absorption via inhibition of pancreatic lipase as this is the major source of excess calories [14]. Orlistat is the derivative of lipstatin, a potent natural inhibitor of pancreatic lipases isolated from bacterium. Orlistat promotes body weight loss and reduces the incidence of diabetes by nearly 40% in obese people [15]. However, it has serious side effects, such as steatorrhea, stomach pain, irregular menstrual periods, and headaches [16].

*Polygonum cuspidatum* (*P. cuspidatum*, Polygonaceae) has been used clinically for the treatment of constipation, gallstones, hepatitis, and inflammation in East Asian countries such as Korea, China, Taiwan, and Japan [17], little work has been carried out regarding the effects on anti-obesity. In the present study, to elucidate activities of *P. cuspidatum* on anti-obesity, we screened candidates for lipase inhibitory activity from the *P. cuspidatum* fractions and investigated the effects of butanol fraction of the ethanol extract of *P. cuspidatum* (POCU1b) on the regulation of adipocyte differentiation. Our findings suggest for the first time that POCU1b inhibits adipocyte differentiation through the attenuation of lipid accumulation.

## Methods

### Preparation of Polygonum cuspidatum extract

Radix of *P. cuspidatum* were purchased from a commercial supplier in Jung-dong, Daejeon, Korea in November 2008 and identified by Prof. J.H. Kim in the Department of Life Science, Gachon University. A voucher specimen was deposited at the Herbarium of Diabetic Complication Research Team, Korea Institute of Oriental Medicine. The dried plant material (5.5 kg) was extracted with ethanol (3 × 36 L) by maceration at room temperature for 3 days and the extracts were concentrated *in vacuo* at 40°C. The concentrated extract (580 g) was diluted in water (2 L) and then partitioned successively with *n*-hexane, ethyl acetate, *n*-butanol, and water, respectively. The percentage (w/w) yield of the lyophilized butanol fraction of the ethanol extract of *P. cuspidatum* (POCU1b) was 1.9%. For water extract, the dried plant (0.1 kg) was extracted with boiling water (3 × 1.5 L) and the extract was *in vacuo* at 40°C. The percentage yield of water extract was 24.8%.

### Pancreatic lipase activity of Polygonum cuspidatum extract and its fractions

The method for measuring pancreatic lipase activity was modified from that of Kim and colleagues [18,19]. Briefly, an enzyme buffer was prepared by the addition of a solution of porcine pancreatic lipase [2.5 mg/ml in 10 mM MOPS (morpholinepropanesulphonic acid) and 1 mM EDTA, pH 6.8] to 169 μl of Tris buffer (100 mM Tris-HC1 and 5 mM CaCl_2_, pH 7.0). Then, either 20 μl of the plant extracts and fractions at the test concentration, or orlistat, was mixed with 20 μl of the enzyme buffer and incubated for 15 min at 37°C with 5 μl of the substrate solution [10 mM p-NPB (p-nitrophenyl butyrate) in dimethyl formamide]. The enzymatic reactions were allowed to proceed for 30 min at 37°C. Lipase activity was determined by measuring the hydrolysis of p-NPB to p-nitrophenol at 405 nm using an ELISA reader (BIO-TEK, Synergy HT, VT, USA).

### Culture and differentiation

The 3 T3-L1 preadipocyte cell line was purchased from the American Type Culture Collection (Rockville, MD, USA). The cells were cultured in 4.5 g/L glucose-DMEM with 10% calf serum, penicillin (100 U/ml), and streptomycin (100 μg/ml) in 10-cm plastic Petri dishes until they reached 100% confluence. For differentiation, 2-day post-confluent cells were incubated for 48 h in DMEM with 10% FBS, antibiotics, and a differentiation cocktail termed MDI, which contained 0.5 mM isobutylmethylxanthine, 1 μM dexamethasone, and 1 μg/ml insulin. After 48 h, the cells were maintained in DMEM with 10% FBS, antibiotics, and 5 μg/ml insulin. Cells were cultured for 12 days in DMEM with 10% FBS and antibiotics, and the media changed every 2 days until the cells were collected for analysis.

### Cytotoxicity assay

Cytotoxicity was evaluated in vitro by determining cell viability using the CCK-8 kit (Cell Counting Kit-8, Dojindo laboratories, Tokyo, Japan). Cells were plated at a density of 1 × 10^3^ cells/ml in 96-well plates and allowed to attach overnight. The cells were then treated with POCU1b (1–200 μg/ml) and incubated for 5 and 12 days. After a 4-h incubation with the CCK-8 solution, absorbance was measured with a microtiter plate reader (Bio-Tek, Winooski, VT, USA) at 450 nm. We calculated the percent viability as optical density of treated sample/optical density of untreated control × 100.

### Oil Red O staining for intracellular triglycerides

Cells were washed twice with PBS on day 12 and fixed on dishes with 3% formaldehyde in PBS for 20 min. After two rinses with PBS, cells were incubated with an Oil red O solution (0.5% Oil red O, 60% isopropanol, and 40% water) and filtered through a 0.22-μm filter for 30 min. The monolayer was extensively washed with water to remove unbound dye. Representative images of treated cells were obtained with an Olympus microscope (BX51, Japan), equipped with an Olympus DP 70 camera. Stained cells were air-dried overnight and then dissolved in isopropanol. Absorbance was measured at 520 nm.

### Glycerol-3-phosphate dehydrogenase (GPDH) activity assay

Treated cell lysates were extracted and used to determine GPDH activity as described [20,21]. Briefly, protein lysate was measured according to the bicinchoninic acid assay (BCA) method, and the GPDH assay was performed to assess the disappearance of NADH during the GPDH-catalyzed reduction of dihydroxyactone phosphate (DHAP) under zero-order conditions as described [20,21].

### Immunoblotting

Immunoblotting was performed using a previously described method [22]. Cells were homogenized in a solution containing 250 mM sucrose, 1 mM ethylenediaminetetraacetic acid (EDTA), 0.1 mM phenylmethylsulfonyl fluoride (PMSF), and 20 mM potassium phosphate buffer (pH 7.6). Equal amounts of protein (25–50 μg/lane) were subjected to immunoblotting with the indicated antibodies. The antibodies used were the following; PPAR-γ (1:1000), adipocyte differentiation-related protein (ADRP, 1:1000), perilipin (1:1,000) from Santa Cruz Biotechnology (Santa Cruz, CA, USA); pAMPK, and C/EBP-α (1:1,000) from Cell Signaling (MA, USA). The bound horseradish peroxidase-conjugated secondary antibody was detected using an enhanced chemiluminescence detection system (iNtRON Biotechnology, SeongNam-Si, Korea). Protein expression levels were determined by analyzing the signals captured on the nitrocellulose membranes using an image analyzer (Las-3000, Fuji photo, Tokyo, Japan).

### RNA extraction and semi-quantitative RT-PCR

Total RNA isolation and RT-PCR were performed as previously described [22]. For RT-PCR, cDNA was synthesized with 1 μg RNA using RT-premix (Bioneer, Daejeon, Korea). The primers used are summarized in Table 1. QuantumRNA 18S (Ambion Inc., Austin, TX, USA) was used as an internal control. PCR products were analyzed by agarose (1.2%) gel electrophoresis along with DNA molecular markers, stained with ethidium bromide, and visualized under UV light. The intensities of RT-PCR products in the agarose gels were quantified by densitometry (Las-3000, Fuji photo).

**Table 1:** Sequences of primers and PCR conditions for semi-quantitative RT-PCR

**Gene**	**Sequence (5′-3′)**	**Annealing temperature (°C)**	**Size (bp)**	**Accession No.**
**ADRP**		55	352	NM_00748
Sense	CTT GTG TCC TCC GCT TAT GTC AGT			
Anti-sense	CTG CTC CTT TGG TCT TAT CCA CCA			
**Perilipin**		55	508	NM_175640
Sense	CTT TCT CGA CAC ACC ATG GAA ACC			
Anti-sense	CCA CGT TAT CCG TAA CAC CCT TCA			
**PPAR-γ**		55	220	NM_011146
Sense	CCA GAG CAT GGT GGG TTC GCT G			
Anti-sense	GAG CTG ACC CAA TGG TTG CTG			
**C/EBP-α**		55	238	NM_007678
Sense	AGG TGG TGG AGT TGA CCA GT			
Anti-sense	CAG CCT AGA GAT CCA GCG AC			

### Statistical analysis

Data are expressed as the mean ± S.E.M. from multiple experiments. Comparison between groups was performed using a one-way ANOVA followed by a Tukey’s test (PRISM software, Graph Pad, San Diego, CA, USA).

## Results

### Lipase inhibitory activity of Polygonum cuspidatum extract and fractions

First, we evaluated in vitro the lipase inhibitory activity of natural products and *P. cuspidatum* was chosen for more detail investigation. Pancreatic lipase inhibition of *P. cuspidatum* extract and fractions is expressed as percentage and IC_50_ (Table 2). Butanol fraction of the ethanol extract of *P. cuspidatum* (POCU1b) exhibited the strongest inhibitory effect on lipase with an IC_50_ value of 15.8 μg/ml. Next, we tested the effect of *P. cuspidatum* extract and fractions on adipocyte differentiation of 3 T3-L1 preadipocytes. POCU1b was the most effective in the prevention of adipogenesis (Additional file 1: Figure S1). Thus, we investigated the anti-obesity effects of POCU1b on adipocyte differentiation of 3 T3-L1 preadipocytes and the related molecular mechanism of lipid accumulation.

**Table 2:** **Lipase inhibitory activity of *Polygonum cuspidatum *** **fractions**

**Scientific name**	**Fraction**	**Conc. (μg/ml)**	**Inhibition (%)**^**a**^	**IC**_**50 **_**(μg/ml)**
*Polygonum cuspidatum*	Ethanol extract (POCU1)	0	0 ± 4.8	72.5 ± 6.7
		50	45.8 ± 1.9	
		100	54.9 ± 0.3	
		150	62.7 ± 3.2	
	*n-*Hexane (POCU1h)	0	0 ± 4.8	326.9 ± 36.6
		200	33.9 ± 3.2	
		300	44.7 ± 7.3	
		400	64.1 ± 7.2	
	Ethyl acetate (POCU1ea)	0	0 ± 4.8	26.4 ± 1.9
		20	44.6 ± 3.4	
		30	52.5 ± 0.7	
		40	54.1 ± 0.4	
	*n-*Butanol (POCU1b)	0	0 ± 4.8	**15.8 ± 2.6**
		10	46.5 ± 0.3	
		15	49.7 ± 2.4	
		20	52.8 ± 1.4	
	Water (POCU1w)	0	0 ± 4.8	295.8 ± 10.9
		200	46.6 ± 1.6	
		300	50.1 ± 0.2	
		400	52.8 ± 0.6	

^a^ Results are the mean ± S.E.M. (n = 3).

### Cytotoxicity assay

**Figure 1:**
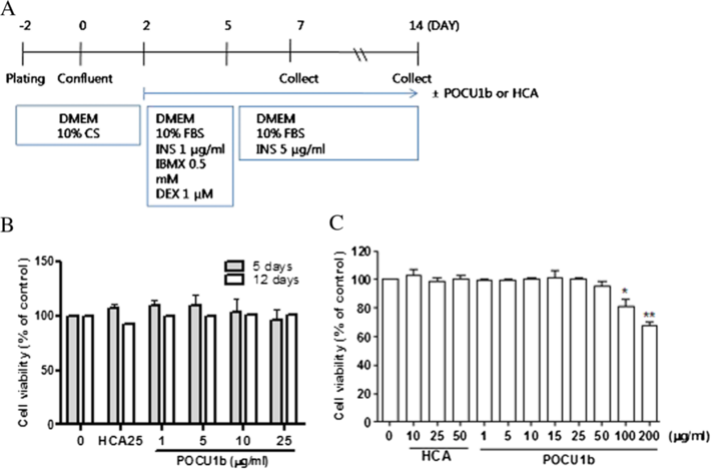
**Cytotoxicity of POCU1b. (A)** 3 T3-L1 differentiation. **(B)** Cytotoxicity of POCU1b using the CCK-8 kit. Cells were treated with different concentrations of POCU1b for 5 or 12 days. Data are expression as the mean ± S.E.M. (n = 4). **(C)** Cytotoxicity at high concentrations of POCU1b. Cells were treated with high concentrations of POCU1b (~200 μg/mL) for 12 days. Data are expression as the mean ± S.E.M. (n = 6).*p < 0.05, **p < 0.01 vs. untreated cells.

Cytotoxicity assay was performed to evaluate whether POCU1b had any effects on 3 T3-L1 viability and to determine the optimal conditions required. During the differentiation processes, 3 T3-L1 preadipocytes were treated with insulin and POCU1b for 5 and 12 days (Figure 1A). There were no effects on cytotoxicity at low concentrations of POCU1b (1, 5, 10, and 25 μg/ml) and HCA (25 μg/ml) (Figure 1B). However, high concentrations of POCU1b (100 and 200 μg/ml) altered cell viability after 12 days (Figure 1C). Hydroxycitric acid (HCA, a positive anti-obesity control) did not alter cytotoxicity after 12 days at concentrations up to 50 μg/ml.

### POCU1b inhibits adipocyte differentiation and GPDH activity

**Figure 2:**
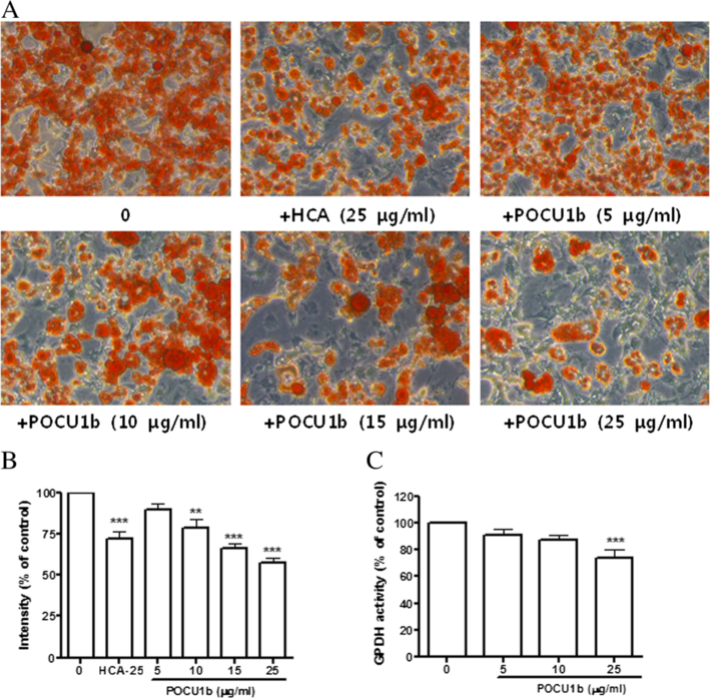
**POCU1b inhibits adipocyte differentiation and GPDH activity. (A)** Oil red O staining for lipid content in 3 T3-L1 adipocytes. 3 T3-L1 preadipocytes were induced to differentiate in POCU1b (5–25 μg/mL) for 12 days. **(B)** Relative density of Oil red O staining. Data are expressed as the mean ± S.E.M. (*n* = 5). **p < 0.01, and ***p < 0.001 vs. untreated cells, respectively. **(C)** Confluent 3 T3-L1 preadipocytes were induced to differentiate in the presence or absence of POCU1b (5, 15, or 25 μg/mL). Cells were lysed on day 12, and GPDH activity was measured. Data are expressed as means ± S.E.M. (*n* = 5) for three independent experiments, normalized to GPDH activity obtained under standard differentiation conditions. ***p < 0.001 vs. untreated cells.

To determine the effects of POCU1b on adipogenic conversion, 3 T3-L1 preadipocytes were induced to differentiate in the presence or absence of POCU1b and stained with Oil red O. Lipid droplets in untreated control cells stained very strongly with Oil red O, an indication that the cells accumulated substantial amounts of cytoplasmic triglycerides. Representative images of Oil red O staining in treated cells showed that POCU1b suppressed both triglyceride accumulation and adipocyte differentiation in a dose-dependent manner (Figure 2A and 2B). HCA treatment of cells also reduced staining with Oil red O, indicative of low triglyceride content.

Expression of GPDH is induced the conversion of preadipocytes to adipocytes [23]. We examined the effect of POCU1b on the activity of GPDH. POCU1b treatment of 3 T3-L1 adipocytes resulted in a marked decrease in GPDH activity in a dose-dependent manner (Figure 2C; POCU1b 25 μg/ml vs. control, *p* < 0.05).

### POCU1b phosphorylates AMP-activated protein kinase (AMPK)

**Figure 3:**
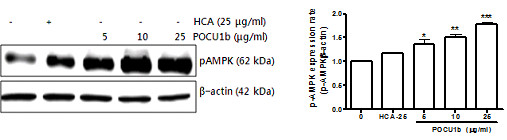
**POCU1b activates AMPK signaling in a dose-dependent manner.** Cells were collected after 12 days and lysed. AMPK activation was determined by immunoblot analysis. Data represent the mean ± S.E.M. of at least three experiments performed in triplicate. *p < 0.05, **p < 0.01, and ***p < 0.001 vs. untreated cells, respectively.

Several studies have suggested that AMPK acts as a major energy sensor and regulator in adipose tissues [24] and that activation of AMPK leads to lipid lowering [25]. To determine the effects of POCU1b on AMPK activation in 3 T3-L1 adipocytes, cells were treated with or without POCU1b and AMPK was detected by immunoblot analysis. Levels of pAMPK were markedly increased in a dose-dependent manner following POCU1b treatment (Figure 3). At a same dose (25 μg/ml), these resulted in a 78% increase for POCU1b and 17% increase for HCA.

### Effect of POCU1b on expression of lipid accumulation-related factors

**Figure 4:**
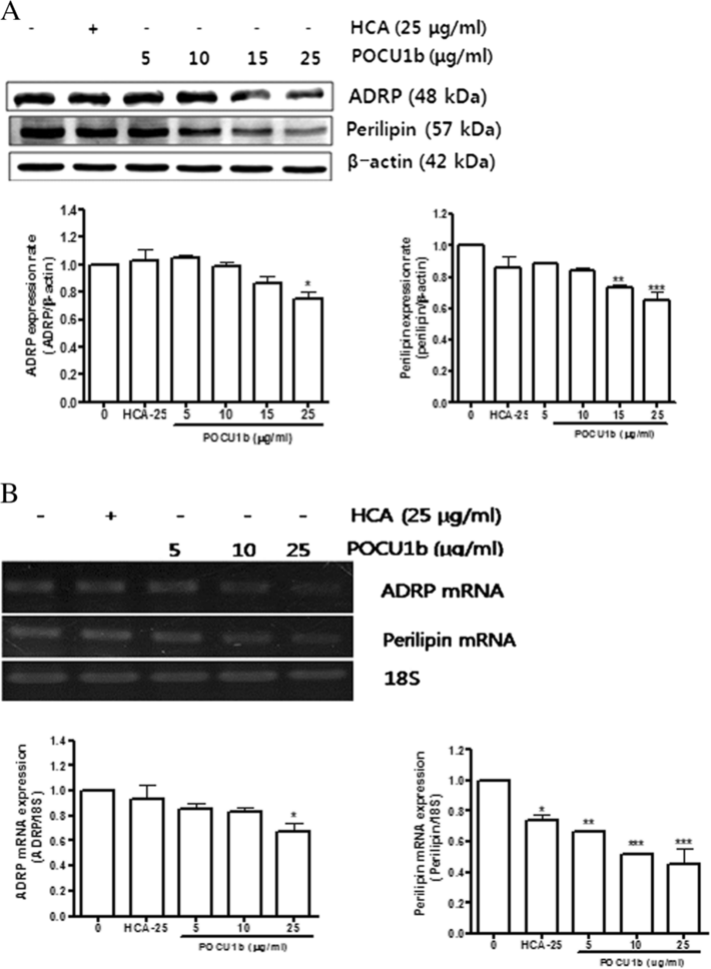
**Effects of POCU1b on expression of ADRP and perilipin expression levels. (A)** ADRP and perilipin protein levels were detected by immunoblot analysis and suppressed in a dose-dependent manner by POCU1b. Data represent the mean ± SEM of at least three experiments performed in triplicate. *p < 0.05, **p < 0.01, and ***p < 0.001 vs. untreated cells, respectively. **(B)** Expressions of ADRP and perilipin mRNA were analyzed by RT-PCR and suppressed in a dose-dependent manner by POCU1b. Data represent the mean ± S.E.M. of at least three experiments performed in triplicate. *p < 0.05, **p < 0.01, and ***p < 0.001 vs. untreated cells, respectively.

ADRP stimulates lipid accumulation and lipid droplet formation [26]. Therefore, we tested the effects of POCU1b on the expression of ADRP, a lipid-associated protein that is expressed during early adipose differentiation. POCU1b reduced protein and mRNA expression levels of ADRP in a dose-dependent manner (Figure 4). Similar to ADRP, perilipin stimulated lipid accumulation and localized to the surfaces of lipid droplets [27,28]. As shown in Figure 4, perilipin protein (Figure 4A, middle panel) and mRNA (Figure 4B, middle panel) expression levels decreased in POCU1b-treated 3 T3-L1 adipocytes in a dose-dependent manner.

### Effect of POCU1b on mRNA and protein expression levels of adipogenic factors

**Figure 5:**
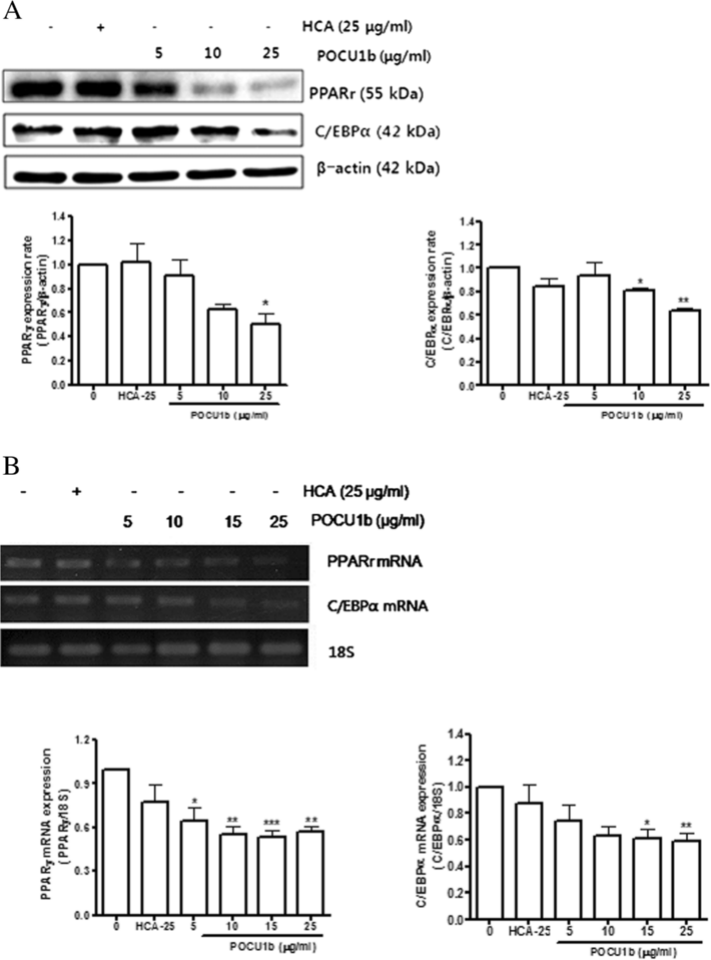
**Effects of POCU1b PPAR-γ and C/EBP-α on protein and mRNA expression levels. (A)** PPAR-γ and C/EBP-α protein levels were detected by immunoblot analysis and suppressed in a dose-dependent manner by POCU1b. Data represent the mean ± SEM of at least three experiments performed in triplicate. *p < 0.05 and **p < 0.01 vs. untreated cells, respectively. **(B)** Expressions of PPAR-γ and C/EBP-α mRNA were analyzed by RT-PCR. Data represent the mean ± SEM of at least three experiments performed in triplicate. *p < 0.05, **p < 0.01, and ***p < 0.001 vs. untreated cells, respectively.

To determine whether suppression of both triglyceride accumulation and GPDH activity was caused by the inhibition of an adipogenic mechanism, we examined the expression levels of mRNA and protein of the adipogenic transcription factors PPAR-γ and C/EBP-α in POCU1b-treated 3 T3-L1 adipocytes. As shown in Figure 5, POCU1b reduced both protein (Figure 5A, upper panel) and mRNA (Figure 5B, upper panel) expression levels of PPAR-γ compared with standard differentiated adipocytes in a dose-dependent manner. Both protein and mRNA expression levels of C/EBP-α were also decreased in a dose-dependent manner in POCU1b-treated 3 T3-L1 cells (Figure 5A and B, middle panel). Thus, we conclude that POCU1b regulates the production of adipogenic transcription factors during the adipocyte differentiation process in 3 T3-L1 adipocytes.

## Discussion

In the present study, POCU1b exhibited the strongest inhibitory effect on lipase activity and the putative anti-obesity effects of POCU1b were investigated for the first time through the inhibition of adipocyte differentiation. POCU1b suppressed triglyceride accumulation in 3 T3-L1 adipocytes. GPDH activity (a marker of adipocyte differentiation) was also significantly diminished by POCU1b in a dose-dependent manner, and pAMPK activity was markedly increased in 3 T3-L1 adipocytes. Furthermore, both ADRP and perilipin levels were decreased, and levels of the adipogenic transcription factors PPAR-γ and C/EBP-α were also significantly decreased after POCU1b treatment. We also observed reduced lipid content in adipocytes, increased phosphorylation of AMPK, inhibition of lipid accumulation via lower ADRP and perilipin levels, and the inhibition of differentiation in 3 T3-L1 adipocytes.

Natural products have been commonly used as dietary supplements to control body weight [13,14]. *P. cuspidatum* has also been used as a natural laxative in traditional Asian herbal medicines [17]. In our study, the ethanol extract of *P. cuspidatum* exhibited strong anti-lipase activity, with an IC_50_ value of 72.5 ±67 μg/ml. Especially, POCU1b showed the strongest activity (IC_50_ = 15.8 ± 1.9 μg/ml). *P. cuspidatum* is composed of phytochemicals such as emodin and resveratrol [17,29,30]. The anti-diabetic effects of emodin and resveratrol have been studied both *in vitro* and *in vivo*[12,31,32]. A recent study showed that emodin exhibits a very high-binding affinity to PPAR-γ and induces both a time- and dose-dependent increase in glucose uptake as well as in GLUT1 and GLUT4 mRNA expression levels in differentiated 3 T3-L1 adipocytes [33].

Adipocytes play a critical role in the regulation of energy balance. Adipose tissue growth involves an increase in adipocyte size and/or number. Changes in adipocyte number are achieved through a complex interplay between the proliferation and differentiation of preadipocytes. Adipocyte differentiation (adipogenesis) regulates the amount of white adipose tissue (WAT) mass [34]. In this study, POCU1b inhibited adipocyte differentiation in 3 T3-L1 cells in a dose-dependent manner. It is noteworthy that inhibition of adipocyte differentiation reduces adipocyte number in WAT, which acts as a secretory/endocrine organ that mediates numerous physiological and pathological processes.

GPDH activity is high in mature adipocytes; the activity of this enzyme is therefore routinely measured to assess adipogenic differentiation in cultured cells [35] and has been used as an index for monitoring triglyceride synthesis [21]. In the current study, we found that treatment with POCU1b (25 μg/ml) suppressed GPDH activity without affecting cell viability. However, treatment with HCA, an active compound of *Garcinia cambogia*, had no significant effects on cells after 12 days (Data not shown). Hasegawa *et al.* showed consistently that GPDH activity was not significantly inhibited by treatment with *Garcinia* extract after 21 days. However, the accumulation of lipid droplets was inhibited [36]. AMPK is activated when cellular energy stores are depleted and accelerates ATP-generating catabolic pathways, including glucose and fatty acid oxidation, while reducing ATP-consuming anabolic pathways, including fatty acid and triacylglycerol synthesis [12]. POCU1b activated AMPK in a dose-dependent manner, suggesting that the reduction in energy storage and the increase in energy production occurred through a change in the intracellular ATP-to-AMP ratio (Figure 3).

Proteins on the surfaces of lipid droplets in adipocytes, especially ADRP and perilipin, serve as nucleation centers for the assembly of lipids into nascent lipid droplets [31,37]. Expression of ADRP and perilipin was also inhibited by POCU1b treatment in 3 T3-L1 adipocytes. POCU1b also blocked the expression of the adipogenic transcription factors C/EBP-α and PPAR-γ, shown to be important players in adipocyte differentiation. C/EBP-α knock-out (C/EBPα −/−) mice neither develop adipose tissue normally nor accumulate triglycerides, the hallmark of WAT, suggesting a central role for C/EBP-α in adipogenesis [38]. Additionally, PPAR-γ is known to be a key protein that is expressed prior to C/EBP-α expression during early adipocyte differentiation of 3 T3-L1 cells [39].

## Conclusions

The present study demonstrated that POCU1b blocks 3 T3-L1 adipocyte differentiation in a dose-dependent manner and inhibits the expression of the key transcription factors PPAR-γ and C/EBP-α. This study also showed that POCU1b treatment prevents the expression of ADRP and perilipin by both attenuating lipid accumulation and activating AMPK phosphorylation. Thus, POCU1b is worthwhile to further investigate for its potential pharmacological effect in metabolic disorders, specifically obesity.

## Competing interests

The authors declare that they have no competing interests.

## Authors’ contributions

YSK and JSK: Designed the study and wrote the manuscript; YSK and YML: Carried out the cell culture experiments; JHK: Identification of plant. All authors read and approved the final manuscript.

## Pre-publication history

The pre-publication history for this paper can be accessed here:

http://www.biomedcentral.com/1472-6882/13/282/prepub

## Supplementary Material

Additional file 1: Figure S1 POCU1 inhibits adipocyte differentiation. Oil red O staining for lipid content in 3 T3-L1 adipocytes. 3 T3-L1 preadipocytes were induced to differentiate in extract (25 μg/mL) or fractions (25 μg/mL) for 12 days. POCU1, the ethanol extract of *P. cuspidatum;* POCU1h, n-hexane fraction of the ethanol extract of *P. cuspidatum*; POCU1ea, ethyl acetate fraction of the ethanol extract of *P. cuspidatum*; POCU1b, n-butanol fraction of the ethanol extract of *P. cuspidatum*; POCU1w, Water fraction of the ethanol extract of *P. cuspidatum. * Click here for file
