# Supplementary figures and images for: Polygonum cuspidatum inhibits pancreatic lipase activity and adipogenesis via attenuation of lipid accumulation
Source: BMC Complement Altern Med. 2013 Oct 25;13:282. doi: 10.1186/1472-6882-13-282 (PMC3819703; doi:10.1186/1472-6882-13-282)

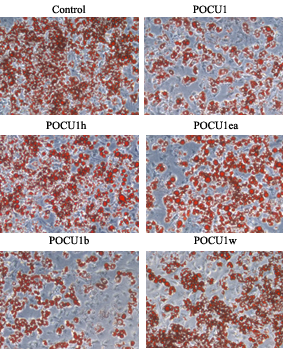

Supplement: Additional file 1: Figure S1 — POCU1 inhibits adipocyte differentiation. Oil red O staining for lipid content in 3 T3-L1 adipocytes. 3 T3-L1 preadipocytes were induced to differentiate in extract (25 μg/mL) or fractions (25 μg/mL) for 12 days. POCU1, the ethanol extract of P. cuspidatum; POCU1h, n-hexane fraction of the ethanol extract of P. cuspidatum; POCU1ea, ethyl acetate fraction of the ethanol extract of P. cuspidatum; POCU1b, n-butanol fraction of the ethanol extract of P. cuspidatum; POCU1w, Water fraction of the ethanol extract of P. cuspidatum. [file 1472-6882-13-282-S1.tiff]
